# ﻿Surveying lichen diversity in forests: A comparison of expert mapping and eDNA metabarcoding of bark surfaces

**DOI:** 10.3897/mycokeys.106.117540

**Published:** 2024-06-21

**Authors:** Lukas Dreyling, Steffen Boch, H. Thorsten Lumbsch, Imke Schmitt

**Affiliations:** 1 Senckenberg Biodiversity and Climate Research Centre (SBiK-F), Frankfurt am Main, Germany Senckenberg Biodiversity and Climate Research Centre (SBiK-F) Frankfurt am Main Germany; 2 Goethe University Frankfurt, Institute of Ecology, Evolution and Diversity, Frankfurt am Main, Germany Goethe University Frankfurt Frankfurt am Main Germany; 3 WSL Swiss Federal Institute for Forest, Snow and Landscape Research, Birmensdorf, Switzerland WSL Swiss Federal Institute for Forest, Snow and Landscape Research Birmensdorf Switzerland; 4 Collections, Conservation, and Research, The Field Museum, Chicago, IL 60605-2496, USA The Field Museum Chicago United States of America

**Keywords:** Assessment, biodiversity, bioindicators, conservation, databases, floristic survey, identification, inventory, metabarcoding, monitoring

## Abstract

Lichens are an important part of forest ecosystems, contributing to forest biodiversity, the formation of micro-niches and nutrient cycling. Assessing the diversity of lichenised fungi in complex ecosystems, such as forests, requires time and substantial skills in collecting and identifying lichens. The completeness of inventories thus largely depends on the expertise of the collector, time available for the survey and size of the studied area. Molecular methods of surveying biodiversity hold the promise to overcome these challenges. DNA barcoding of individual lichen specimens and bulk collections is already being applied; however, eDNA methods have not yet been evaluated as a tool for lichen surveys. Here, we assess which species of lichenised fungi can be detected in eDNA swabbed from bark surfaces of living trees in central European forests. We compare our findings to an expert floristic survey carried out in the same plots about a decade earlier. In total, we studied 150 plots located in three study regions across Germany. In each plot, we took one composite sample based on six trees, belonging to the species *Fagussylvatica*, *Piceaabies* and *Pinussylvestris*. The eDNA method yielded 123 species, the floristic survey 87. The total number of species found with both methods was 167, of which 48% were detected only in eDNA, 26% only in the floristic survey and 26% in both methods. The eDNA contained a higher diversity of inconspicuous species. Many prevalent taxa reported in the floristic survey could not be found in the eDNA due to gaps in molecular reference databases. We conclude that, currently, eDNA has merit as a complementary tool to monitor lichen biodiversity at large scales, but cannot be used on its own. We advocate for the further development of specialised and more complete databases.

## ﻿Introduction

Lichens are important components of biodiversity in forest ecosystems, where they form epiphytic communities in the canopy (Ellis 2012) and on tree trunks (Hofmeister et al. 2016). In central European forests, lichens and their symbionts are characteristic taxa of the bark surface community (Baldrian 2017; Dreyling et al. 2022; Hofmann et al. 2023). In fact, the bark of trees has been proposed as an important part of the forest microbiome and sustains a high microbial biomass especially if lichens are present (Baldrian 2017). Temperate forests harbour more than a hundred, often several hundred species of lichen-forming fungi (Jüriado et al. 2003; Coppins and Coppins 2005; Boch et al. 2013; Lõhmus and Lõhmus 2019). The lichen communities contribute to forest ecosystem function by retaining water (Van Stan and Pypker 2015), cycling minerals and nutrients (Pike 1978; Reiners and Olson 1984; Knops et al. 1991, 1996; Campbell et al. 2010), being part of the food web and providing habitat and micro-niches for other organisms (reviewed in Ellis (2012) and Asplund and Wardle (2017)). Forest lichen communities respond to abiotic environmental changes (Miller et al. 2018; Łubek et al. 2021), as well as to forest management (Nascimbene et al. 2013; Boch et al. 2021). Some species can be used as indicators to monitor the effects of anthropogenic pollutants (Frati and Brunialti 2023). These are important reasons to survey and monitor lichen biodiversity in forests.

The assessment of lichen biodiversity can be challenging, even for taxonomic experts (Vondrák et al. 2016). Since species identification of lichenised fungi often relies only on few morphological characters (Crespo and Lumbsch 2010), considerable expertise is necessary and often requires specimen collection for ex situ identification, for example, through microscopy or chemical tests (Wright et al. 2019). As a result, the outcomes of lichen surveys are highly dependent on the training of collectors (Giordani et al. 2009). Additionally, lichen-forming fungi are a group with high potential for cryptic diversity that cannot be distinguished in the field (Crespo and Lumbsch 2010; Altermann et al. 2014).

Molecular markers are useful complementary tools to aid the identification of lichenised and non-lichenised fungi (Lücking et al. 2020). Especially, the ITS barcode marker is increasingly used for species identification and species delimitation of lichenised fungi (Schoch et al. 2012; Bradshaw et al. 2020). It has been applied to assess species diversity within geographic regions (Kelly et al. 2011), as well as within taxonomic groups, for example, Parmeliaceae (Divakar et al. 2016). While ITS barcoding works well for the majority of species, it has limitations in some taxonomic groups, for example, the genus *Cladonia*, which seems to be lacking a sufficient barcode gap at least in some species complexes (Pino-Bodas et al. 2013; Marthinsen et al. 2019) or members of Graphidaceae and Pertusariaceae, which do not amplify reliably with common ITS primers, so that their ITS is not used in multi-locus phylogenies (Schmitt and Lumbsch 2004; Rivas Plata et al. 2013) and they remain under-represented in DNA databases. A small number of studies have attempted to identify species and characterise the lichen community by metabarcoding bulk specimen collections (Wright et al. 2019; Henrie et al. 2022). They found that this method produces comparable results between minimally trained and expert collectors and thus potentially reduces the need for extensive training (Wright et al. 2019; Henrie et al. 2022). Furthermore, they concluded that metabarcoding surveys could enable a more efficient sampling over a larger spatial extent (Wright et al. 2019).

Biodiversity assessments using environmental DNA (eDNA) allow species-level identification from DNA present in environmental samples, such as water, soil or air (Taberlet et al. 2012; Yoccoz 2012). In comparison to bulk metabarcoding, this method has additional advantages, such as targeting a broader range of taxa (Taberlet et al. 2012) and being non-invasive, i.e. not requiring destruction of specimens (Deiner et al. 2017). Despite some drawbacks, in particular due to incomplete databases and primer bias (Bellemain et al. 2010; Keck et al. 2022), eDNA has shown great potential for biodiversity assessments (Shirouzu et al. 2016; Frøslev et al. 2019). When eDNA and conventional methods were compared, species overlap was variable depending on the taxonomic group, but eDNA always identified taxa that were not picked up with other methods (Cordier et al. 2021). In a meta-analysis, eDNA was found to detect more species in general and significantly more rare species, exhibiting higher accuracy and efficiency, while being less costly than conventional biodiversity assessments (Fediajevaite et al. 2021).

In this study, we analyse the utility of eDNA – obtained from bark surfaces of tree trunks at breast height – to assess the diversity of lichen communities in central European forests. In previous studies, we have generated datasets of entire fungal communities associated with bark surfaces, based on ITS metabarcoding (Dreyling et al. 2022, 2024; Hofmann et al. 2023). Here, we use only the fraction of lichenised fungi from these datasets. The sampling sites are 150 specific plots located within the Biodiversity Exploratories (Fischer et al. 2010) in northern, central and southern Germany. We compare the results to a previous floristic survey carried out in the same plots (Boch et al. 2021). Specifically, we address the following questions: I. Which species of lichenised fungi can be identified from environmental samples via eDNA metabarcoding? II. What are the differences to the diversity obtained through an expert survey? III. Is eDNA metabarcoding a reliable tool to survey lichen diversity in forests?

## ﻿Material and methods

### ﻿Study sites

We surveyed communities of forest-dwelling lichen species in 150 plots, located in three regions, within the Biodiversity Exploratories framework (Fischer et al. 2010). The regions mark a south-west to north-east gradient across Germany and differ in their climate and topography. The plots within the regions were further selected along a gradient of the anthropogenic impact, i.e. forest management intensity (Fischer et al. 2010; Boch et al. 2021) and are representative for central European forest ecosystems. The South-West region is on average 2 °C colder than the North-East (6.5 °C vs. 8.5 °C) and experiences approximately twice the amount of precipitation (700–1000 mm vs. 500–600 mm; Fischer et al. (2010)). In addition, they differ in their tree species composition, with beech trees (*Fagussylvatica*) as the dominant species in most plots across the regions. Some plots are dominated by coniferous trees, specifically Norway spruce (*Piceaabies*) in the South-West region and Scots pine (*Pinussylvestris*) in the North-East region (Schall and Ammer 2018). For both, eDNA sampling and classical lichen mapping, we surveyed a 20 m × 20 m subplot within the established 100 m × 100 m experimental plots.

### ﻿eDNA sampling and processing

**Figure 1.**
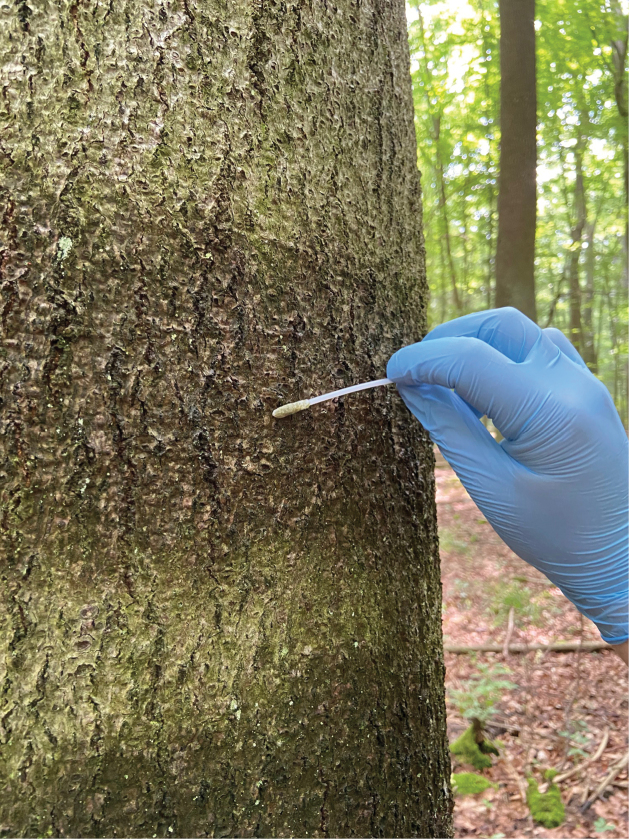
Sampling procedure: We moistened the tree trunk on all sides at breast height and swabbed the bark surface in a zigzagging motion along a 10 cm wide band around the tree trunk. The swabbed area included smooth bark surfaces and crevices, as well as epiphytic organisms, if they were present.

In each plot, we collected eDNA samples from the bark surface of six trees of the respective dominant species in May 2021. The six individual tree samples were pooled into one composite sample per plot. Since we had previously shown large community differences between tree sizes (Dreyling et al. 2022), we included two trees each of large (> 30 cm diameter at 150 cm height), medium (15–30 cm) and small (5–15 cm) size in each sample. If this type of sampling was not possible, we included additional trees of the size class that best represented the forest in the immediate surrounding. To sample the bark surface eDNA, we moistened the bark surface with sterile water and then used a nylon-flocked medical swab (FLOQSwabs™, Copan, Brescia, Italy) to collect the bark surface biofilm (Fig. 1). We swabbed around the full tree trunk at approximately 150 cm height from the forest floor, excluding large patches of bryophytes to avoid bias due to the amplification of plant ITS, but explicitly including all other epiphytic organisms. The material collected with the swabs was fixed with 5 ml nucleic acid preservation (NAP) buffer (Camacho-Sanchez et al. 2013) in 15 ml tubes directly after sampling and placed on ice in the field. Afterwards, the samples were stored at 4 °C until DNA extraction in the following week.

A detailed description of the DNA extraction and bioinformatic processing of sequencing reads is given in Dreyling et al. (2022). In brief: We extracted DNA, from samples as well as three extraction blanks, using an extraction kit (Quick-DNA Fecal/Soil Microbe Microprep, Zymo Research Europe GmbH, Freiburg, Germany) with an additional step ensuring liberation of material from the swab. Targeting the ITS2 region, we subsequently amplified the fungal DNA in triplicate, using the universal primer pair fITS7 (GTGARTCATCGAATCTTTG) (Ihrmark et al. 2012) and ITS4 (TCCTCCGCTTATTGATATGC) (White et al. 1990). PCR reactions also included negative controls (without sample material) and multiplex controls (empty wells). We cleaned the amplicons via a magnetic bead protocol (MagSI-NGSPREP Plus, magtivio B.V., Geelen, Netherlands) and measured DNA concentration through fluorometry (Qubit dsDNA HS assay on a Qubit 3.0, Thermo Fisher Scientific, MA, United States), before equimolar pooling. The library preparation and Illumina sequencing (MiSeq 2 × 300 bp paired-end) was carried out by Fasteris SA (Plan-les-Ouates, Switzerland) according to their MetaFast Protocol, designed to avoid additional PCR bias.

We used Cutadapt (v3.3; Martin (2011)) to demultiplex the obtained sequencing reads and DADA2 (Callahan et al. 2016) to infer Amplicon Sequencing Variants (ASVs). Taxonomy was assigned against the Martin7 database (Vondrák et al. 2023) using a local BLAST (Altschul et al. 1990) search. Assignments were kept if the “percent identity” was higher than 97%. Additionally, we used the UNITE database (Abarenkov et al. 2022, Version 9.0, incl. non-fungal eukaryotic DNA as outgroups) and the NCBI nucleotide database (Sayers et al. 2022, percent identity > 97%) to assign additional taxonomy to the ASVs that could not be assigned with the Martin7 database. We used FUNGuild (Nguyen et al. 2016) to assign information on the functional guild to the additionally assigned fungal ASVs and filtered the dataset to only contain ASVs which were classified as lichenised fungi. All scripts on the bioinformatic processing, as well as the analysis, are available at Github at https://github.com/LukDrey/eDNA_lichen_survey.

### ﻿Traditional floristic survey

The floristic survey was carried out in 2007 and 2008 and recorded occurrences of lichenised fungi in over 600 plots of the Biodiversity Exploratories (Boch et al. 2021), including the 150 plots of the eDNA sampling. The survey covered a comparable area of 20 m × 20 m around the plot centre, which was not fully identical, but always spatially close to the sampling area of the eDNA survey. All lichens occurring on bark (up to 2.5 m height), rocks, deadwood and soil were recorded. We did not limit the survey time per plot due to strongly varying environmental heterogeneity amongst plots. Most specimens were identified in situ, except when microscopic or chemical characters had to be assessed.

### ﻿Comparison of the two methods

A number of taxonomic changes took place in the approximately 13 years between the two surveys. We accommodated for these developments by harmonising the two species lists and adopting the names accepted as the current names in MycoBank (Crous et al. 2004; Robert et al. 2013). Additionally, we only included species from the floristic dataset, which were recorded as epiphytes in the 150 experimental plots, thus excluding species collected from rocks, deadwood and soil. A list of all species is provided as Suppl. material 1.

To allow for comparisons between the two methods, we transformed the read counts obtained through the eDNA metabarcoding to presence-absence data. Using the two presence-absence datasets, we compared the two methods and assessed the diversity and species richness found with each method. Furthermore, we calculated the number of plots in which a species was found. Finally, we selected five species to visualise geographical occurrence patterns, based on the two different assessment methods.

## ﻿Results and discussion

**Figure 2.**
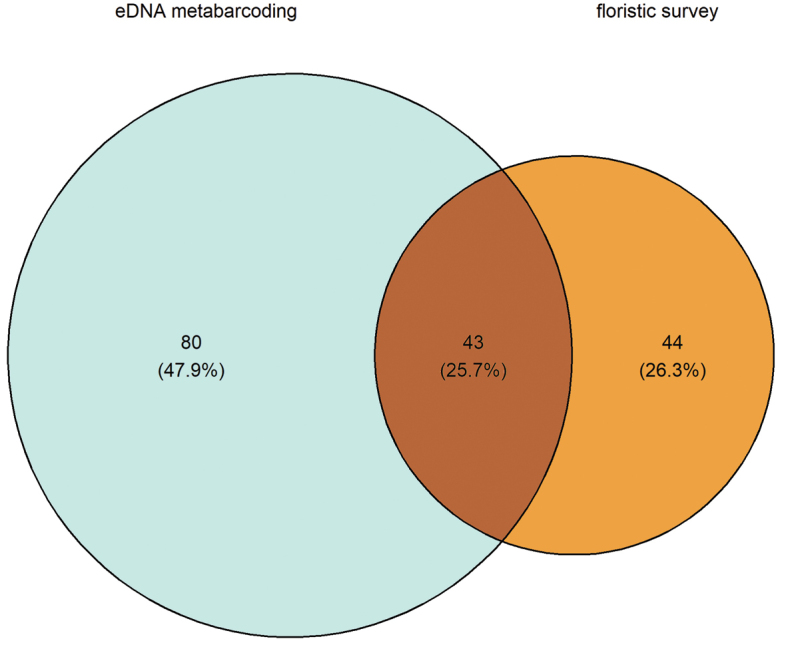
Number and proportion of species of lichenised fungi found either in eDNA metabarcoding or floristic survey or in both methods.

**Figure 3.**
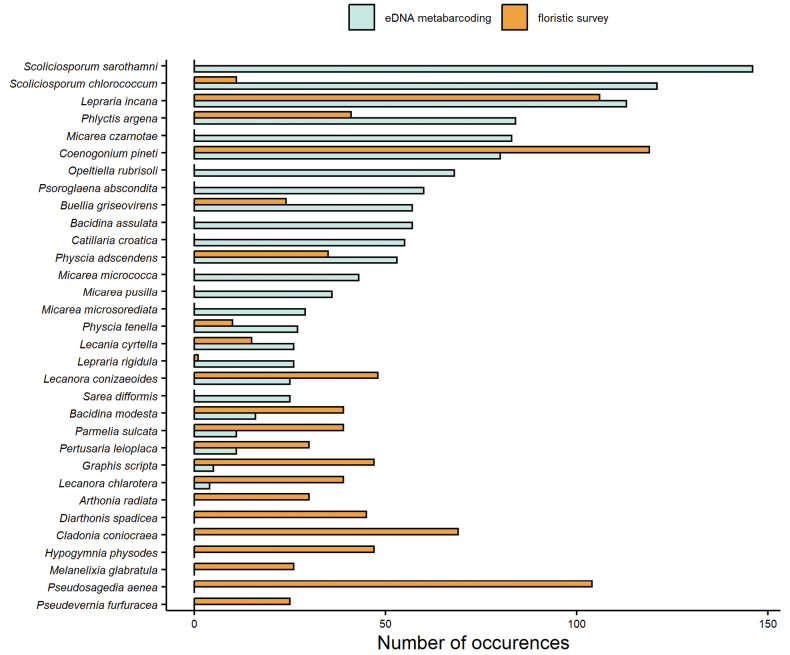
Most common species of lichenised fungi detected by either method (eDNA metabarcoding or floristic survey). We show taxa, which occurred in at least 25 plots (out of 150) across the three regions.

In total, we found 167 species of lichenised fungi in the two surveys. The eDNA method found 123 species, while the traditional floristic survey recorded 87 species (Fig. 2). With the eDNA method, we found 80 species that were not found via the traditional survey methods, while with the floristic survey, we found 44 species not detected by the eDNA method. The higher number of taxa identified from the eDNA is congruent with bulk-specimen sequencing studies from other ecosystems (Wright et al. 2019). Interestingly, only 26% of the total taxa were shared between both methods, likely due to the number of small, inconspicuous genera, such as *Micarea* (Launis et al. 2019), that were only found with the eDNA (Fig. 3). The overlap between the two methods is similar to what has previously been reported for comparisons of eDNA to fruiting body surveys of forest fungi (Shirouzu et al. 2016; Frøslev et al. 2019).

In our study, several species are detected exclusively or predominantly by either of the two methods. Additionally, even the most common species are not necessarily detected by both methods. For example, out of the five most common species (Fig. 3), two were not found in the traditional floristic study, *Scoliciosporumsarothamni* and *Micareaczarnotae*. This result is especially striking for *S.sarothamni* which was found in 146 of 150 plots via eDNA metabarcoding, but was not identified in the floristic study. This species is very small and thus hard to find and distinguish (Kowalewska and Kukwa 2003; Dymytrova 2011). Therefore, it is plausible that it has been overlooked or simply grouped with other taxa, such as its sister species *Scoliciosporumchlorococcum*, in the floristic survey. However, *S.chlorococcum* was also highly prevalent in the eDNA study (121 occurrences), but rarely found in the floristic dataset (11 occurrences). A potential reason for *Scoliciosporum* being less prevalent in the floristic dataset is that both species have a greenish thallus and are often occurring within dominant green algal colonies, making them hard to recognise with traditional methods, especially when sterile.

Other taxa, commonly found in the eDNA metabarcoding dataset, had not been formally described at the time of the floristic survey. For example, both *Opeltiellarubrisoli* and *Micareaczarnotae* were only described in 2019 (Launis et al. 2019; Liu et al. 2019). *Micareaczarnotae* had previously been included in *M.prasina*, which was also only found in eight plots in the floristic study. In general, the eDNA dataset contained a high number of inconspicuous taxa from genera that are difficult to distinguish, such as *Micarea* (Launis et al. 2019), *Scoliciosporum* (Dymytrova 2011) and *Bacidina* (Czarnota and Guzow-Krzemińska 2018). Consistent with our findings, other studies have previously reported that eDNA was superior in revealing hidden diversity for fungi (Shirouzu et al. 2016), including lichen-forming fungi (Wright et al. 2019). An additional advantage of the eDNA approach might be the detection of taxa not directly occurring on the sampled substrate itself, for example, from propagules (Wright et al. 2019; Henrie et al. 2022).

The floristic dataset also includes numerous taxa which were not identified in the eDNA approach. For example, *Pseudosagediaaenea*, a common species found in the floristic survey (occurring in 104 plots), was not found by the eDNA metabarcoding (Fig. 3), albeit ITS sequences of this taxon are included in the sequence repositories used in this study. One potential reason is that their habitat is outside of the sampled area, for example, in the tree crowns or at the base of the tree. In fact, several species prevalent in the floristic dataset, but not the eDNA, occur in these habitats, for example, *Cladoniaconiocraea* at the base of trees (Wirth et al. 2013), *Pseudeverniafurfuracea* on branches in the canopy (Kranner et al. 2003) and also *P.aenea* at the stem base (Larsen et al. 2020). Therefore, restricting the eDNA sampling, or any survey, to a single forest substrate is likely insufficient to describe the full lichen diversity (see also Boch et al. (2013)).

Overall, only very few species were found in a similar number of plots with both methods (Fig. 3). The most prevalent species found with both methods were *Coenogoniumpineti* and *Leprariaincana*. *Coenogoniumpineti* was found in 119 plots in the floristic study and in 80 plots in the eDNA dataset. The prevalence of *L.incana* was even more similar, being found in 106 plots with floristic and 113 plots with eDNA methods. Although, both species preferentially grow at the base of trees (Lackovičová and Guttová 2005; Larsen et al. 2020), they were also often recorded with the eDNA method. It is possible that the dispersal units of these taxa (ascospores in *C.pineti* and soredia in *L.incana*) are dispersed further up the stem, for example, by wind. Furthermore, snails and slugs may play a role in distributing lichen propagules along the stem (Asplund et al. 2010; Boch et al. 2011).

Another apparent reason for the differences in eDNA and floristic surveys are related to the databases necessary for taxonomic assignment of the metabarcoding reads. Despite large efforts in recent years towards the development of reference databases for fungal taxonomy, like the UNITE database (Abarenkov et al. 2023) or the GlobalFungi project (Větrovský et al. 2020), many gaps remain. In our study, several species, commonly found in the floristic study, have no reference sequences in the UNITE database, including *Diarthonisspadicea* and all species of the genus *Arthonia*. Previous studies have proposed to close the gaps in the reference databases by large scale sequencing of lichen herbarium specimens (Gueidan and Li 2022). Regional databases, for example, for Great Britain and Ireland (Kelly et al. 2011) or part of the western USA (Kerr and Leavitt 2023) were helpful in identifying lichen specimens, based on barcodes or bulk metabarcoding. The recently-published Martin7 database, focusing on central European lichens (Vondrák et al. 2023), greatly improved the results of the present study. It enabled the assignment of taxonomy to over 30 additional ASVs, resulting in 27 additional species compared to an initial assignment using the UNITE database.

Technical issues related to sequencing might be the reason that some species present in the floristic study could not be found in the eDNA assessment, although the ITS sequences are included in the UNITE and Martin7 databases. A search with Primer-BLAST (Ye et al. 2012) revealed that, in some cases, the primer combination used in this study could likely not amplify these species. However, some species, commonly occurring in the floristic study such as *Hypogymniaphysodes* or *Pseudeverniafurfuracea*, should have been amplified with the current primers, indicating other issues. A potential reason might be PCR biases influencing which taxa or groups are preferentially amplified (Bellemain et al. 2010). For example, shorter DNA fragments are usually amplified more often (Deagle et al. 2006). It is possible that we missed ITS sequences that are longer because they contain introns, a frequent and stochastic feature of the rDNAs of lichen-forming fungi (Simon et al. 2005). Furthermore, the output of the sequencing machine is limited, so that taxa with few copies might not be sequenced (Gloor et al. 2017).

There is a temporal gap of approximately 13 years between the floristic survey and the eDNA sampling, which may explain some of the observed differences, especially with regard to pollution with sulphur dioxide and nitrogen. Sulphur dioxide (SO_2_) pollution has been decreasing in western Europe since the 1970s, enabling the return of many species to formerly uninhabitable ecosystems (Rose and Hawksworth 1981; Nash and Gries 2002). Conversely, species that are tolerant to acidic and sulphur-enriched conditions, for example, *Lecanoraconizaeoides*, have been reported to decline in central Europe (Nash and Gries 2002; Farkas et al. 2022). In our study, the number of plots, in which *L.conizaeoides* was identified with eDNA in 2021, has halved in relation to the floristic study in 2007/2008. Today, nitrogen pollution is more important in shaping lichen communities than SO_2_ pollution (Purvis et al. 2003; Hultengren et al. 2004; Pinho et al. 2008; Gadsdon et al. 2010). Temperate forests experience increased deposition of nitrogen, for example, through ammonia fertilisers or nitric oxides from fuel combustion, and nitrophytic species have increased in the recent past (Carter et al. 2017). In the present study, two species regarded as nitrophytes, *Physciaadscendens* and *P.tenella* (Gadsdon et al. 2010), have been found more frequently in the eDNA sampling than in the earlier floristic survey (Fig. 3). Interestingly, other nitrophytic species, such as *Xanthoriaparietina*, *Phaeophysciaorbicularis* or *Candelariellareflexa* (Gadsdon et al. 2010), were found less frequently or not at all in the eDNA sampling (Suppl. material 1). In addition, differences between the floristic and the eDNA survey in lichen diversity and community composition might be because of successional developments or the disruption of such developments by forestry management, leading to changes in forest structure and composition, i.e. changed environmental conditions. Such changes might have been even accelerated by climate change that has been proposed to change lichen diversity and community composition (van Herk et al. 2002; Aptroot 2009; Allen and Lendemer 2016; Nascimbene et al. 2016; Nelsen and Lumbsch 2020).

The three study regions differed considerably in their lichen diversity. In the eDNA metabarcoding survey, the proportion of fungal reads assigned to lichens was highest in the south-western region with approximately 39% of the total fungal reads, 27% in the north-eastern and lowest in the central region with only 14%. On average, lichens accounted for 27% of the total fungal reads. We observe a similar pattern in the floristic survey, where the highest number of species was also recorded in the south-western region (82 species), followed by the central and the north-eastern region (32 species). Previous studies in the Biodiversity Exploratories found similar relationships between the regions for plants (Klaus et al. 2013) and arthropods (Simons et al. 2014) that are potentially explained by differences in climate, land-use intensity or nutrient availability. In our study, the higher species richness in the South-West region is likely related to the higher annual precipitation (Fischer et al. 2010), which has been shown to positively influence lichen richness (Marini et al. 2011), but also because of the generally lower former SO_2_ deposition compared to the other two regions (Umweltbundesamt 2005).

**Figure 4.**
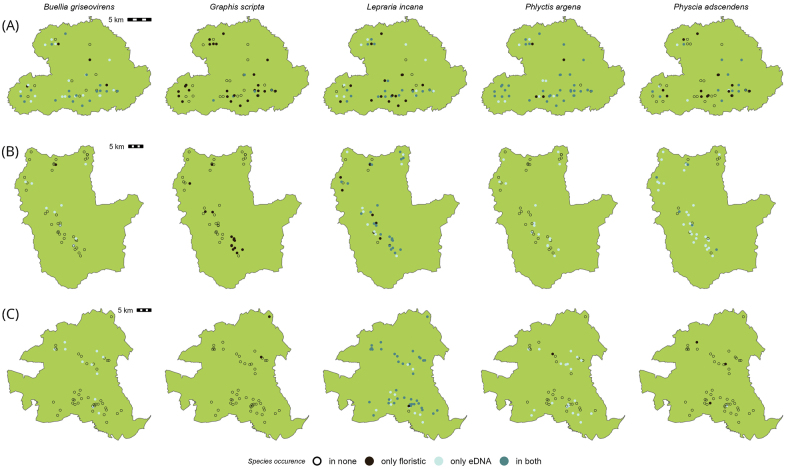
Distribution of five example species within the analysed forest plots of the three regions. Shown are occurrence data based on the floristic survey and eDNA metabarcoding. Each map represents one region (Biodiversity Exploratory) **A** South-West (Swabian Alb) **B** Central (Hainich-Dün) **C** North-East (Schorfheide-Chorin). Each circle depicts a 100 m × 100 m forest plot.

The differences amongst the three study regions are also apparent in the distribution maps of the five example species, *Buelliagriseovirens*, *Graphisscripta*, *Leprariaincana*, *Phlyctisargena* and *Physciaadscendens*. These species were chosen as examples because they were amongst the most prevalent species (Fig. 3) found with both methods, but varied in how often they were recorded. In general, the highest number of plots, in which the example species were recorded, were located in the South-West region (Fig. 4A), while they did not occur in most plots of the North-East region (Fig. 4 C). If a lichen species was frequently recorded by both methods, such as *B.griseovirens*, *L.incana* and *P.argena* in the South-West (Fig. 4A), then it was also found by one of the methods in spatially close plots. In general, the lichen records do not follow a clearly distinguishable pattern of spatial clustering within the regions.

The detection of these five lichen species was different between the methods in the each of the regions. Of the five example species, only *L.incana* was consistently found with both methods across the three regions and consequently is one of the most prevalent species we found. *B.griseovirens* and *P.argena* were found more often in the eDNA samples and almost exclusively with eDNA in the Central and North-East regions (Fig. 4B, C). It is tempting to speculate about a northward shift of the distribution of these species considering the time difference between the two studies, which could explain the absence in the Central and North-East regions during the floristic survey. In addition, the considerably decreased pollution in these two regions might have led to the recovery of lichen communities with many species re-colonising such formerly heavily polluted areas (e.g. Gilbert (1992)).

Nevertheless, *Graphisscripta* was rarely found in the eDNA, but recorded across all three regions in the traditional survey. This pattern is likely related to the use of ITS2 as a molecular marker in the eDNA, which has previously shown low amplification rates for the genus *Graphis* (e.g. Kraichak et al. (2019)). Interestingly, *P.adscendens* was found with both methods in the South-West, purely with the eDNA in the Central and only via floristic survey in the North-East region.

## ﻿Conclusions

In its current form, eDNA metabarcoding cannot be used as a stand-alone tool to survey epiphytic lichen diversity. However, it can serve as a valuable complementary tool, similarly to studies from many other taxonomic groups (Beng and Corlett 2020; Fediajevaite et al. 2021). In the long run, with more correct and more complete ITS databases, we think that the bulk of species from floristic studies can, indeed, be identified with this method. We have to be aware that there are some taxonomic groups, which have too little ITS variability or too little amplification success, to be determined with this tool. A field, which could benefit from metabarcoding of eDNA, is community ecology of lichen-forming fungi, for example, understanding communities and species assemblages of lichenised fungi, their photobiont partners and other thallus-associated microorganisms. For example, species co-occurrences, based on eDNA, could be used to explore the concept of photobiont-mediated guilds (Rikkinen 2003). We have previously shown – based on the same DNA samples used here – that the communities of fungi, green algae and bacteria present on bark surfaces, strongly affect each other’s beta diversities (Dreyling et al. 2024), suggesting that functional guilds, for example, of mycobionts and their photobiont partners, might be also be detected in the present data. Taxonomic assignments need to be carefully examined to assess if assignments are sensible for the geographic region of interest. Looking forward, the recent development of lichen specific databases might solve some of these issues. If eDNA biodiversity assessments are taken beyond the description of diversity, recently developed methods circumvent this issue altogether and are able to use unclassified taxa in the prediction of ecological states (Keck et al. 2023). Future studies of lichen biodiversity could employ these methods and expand the use of lichens as modern biomonitoring agents.

## ﻿Additional information

### Author ORCIDs

Lukas Dreyling https://orcid.org/0000-0001-9839-9504

H. Thorsten Lumbsch https://orcid.org/0000-0003-1512-835X

Imke Schmitt https://orcid.org/0000-0002-7381-0296

### Data availability

The raw sequencing data for this study is provided in the NCBI Sequence Read Archive under accession number SRR23371988. The code for the analysis is available at https://github.com/LukDrey/eDNA_lichen_survey.

## Supplementary materials

Supplementary material 1 List of all species found in this study Data type csv Explanation note The table contains information on wether the species was identified with either the eDNA method or the floristic survey and in how many plots it occured. https://binary.pensoft.net/file/1076436This dataset is made available under the Open Database License (http://opendatacommons.org/licenses/odbl/1.0/). The Open Database License (ODbL) is a license agreement intended to allow users to freely share, modify, and use this Dataset while maintaining this same freedom for others, provided that the original source and author(s) are credited.Lukas Dreyling, Steffen Boch, H. Thorsten Lumbsch, Imke Schmitt
